# Fingolimod for the treatment of neurological diseases—state of play and future perspectives

**DOI:** 10.3389/fncel.2014.00283

**Published:** 2014-09-12

**Authors:** Robert Brunkhorst, Rajkumar Vutukuri, Waltraud Pfeilschifter

**Affiliations:** ^1^Cerebrovascular Research Group, Department of Neurology, Frankfurt University HospitalFrankfurt am Main, Germany; ^2^Institute of General Pharmacology and Toxicology, pharmazentrum frankfurt, Goethe University FrankfurtFrankfurt am Main, Germany

**Keywords:** sphingosine 1-phosphate, ceramides, sphingosine kinase, sphingosine 1-phosphate receptors, multiple sclerosis, stroke, epilepsy, dementia

## Abstract

Sphingolipids are a fascinating class of signaling molecules derived from the membrane lipid sphingomyelin. They show abundant expression in the brain. Complex sphingolipids such as glycosphingolipids (gangliosides and cerebrosides) regulate vesicular transport and lysosomal degradation and their dysregulation can lead to storage diseases with a neurological phenotype. More recently, simple sphingolipids such ceramide, sphingosine and sphingosine 1-phosphate (S1P) were discovered to signal in response to many extracellular stimuli. Forming an intricate signaling network, the balance of these readily interchangeable mediators is decisive for cell fate under stressful conditions. The immunomodulator fingolimod is the prodrug of an S1P receptor agonist. Following receptor activation, the drug leads to downregulation of the S1P_1_ receptor inducing functional antagonism. As the first drug to modulate the sphingolipid signaling pathway, it was marketed in 2010 for the treatment of multiple sclerosis (MS). At that time, immunomodulation was widely accepted as the key mechanism of fingolimod’s efficacy in MS. But given the excellent passage of this lipophilic compound into the brain and its massive brain accumulation as well as the abundant expression of S1P receptors on brain cells, it is conceivable that fingolimod also affects brain cells directly. Indeed, a seminal study showed that the protective effect of fingolimod in experimental autoimmune encephalitis (EAE), a murine MS model, is lost in mice lacking the S1P_1_ receptor on astrocytes, arguing for a specific role of astrocytic S1P signaling in MS. In this review, we discuss the role of sphingolipid mediators and their metabolizing enzymes in neurologic diseases and putative therapeutic strategies arising thereof.

## The sphingolipid signaling pathway

**Figure 1:**
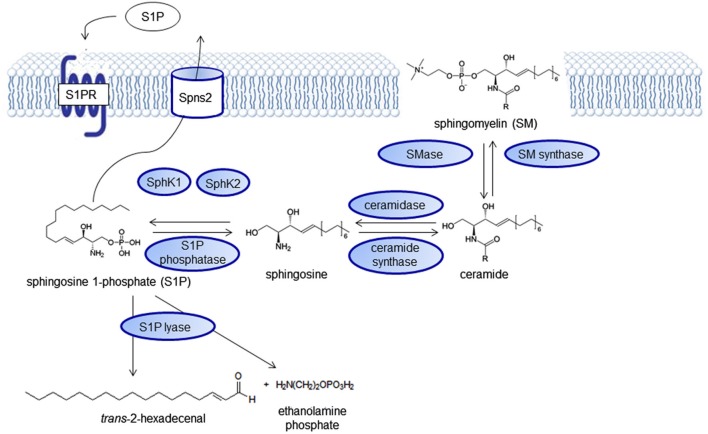
**The sphingolipid signaling pathway**. Sphingolipid signaling molecules are derived from the membrane lipid sphingomyelin and intricately regulated forming a “rheostat” that can be imbalanced in disease states.

**Figure 2:**
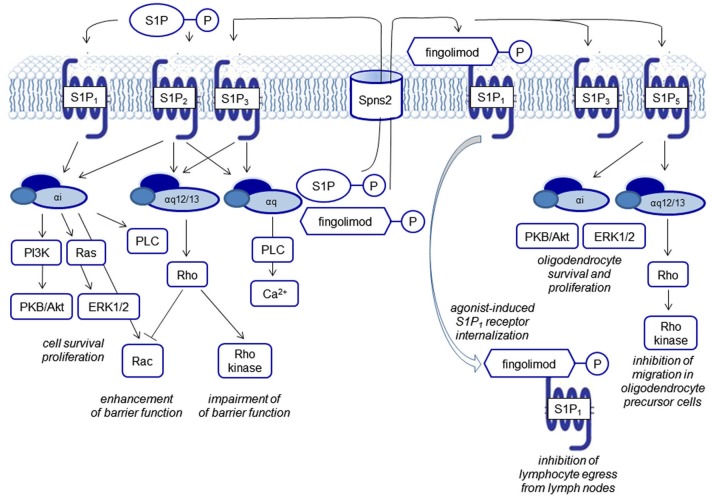
**Biological functions of S1P receptor signaling**. S1P signals via five G protein-coupled receptors, termed S1P_1–5_. Each of the receptors couples to several G proteins eliciting different downstream events. FTY720 phosphate activates all S1P receptors except S1P_2_. S1P and FTY720 phosphate are generated by intracellular phosphorylation of sphingosine and FTY270, respectively and transported outwards to elicit signaling via S1P receptors. Characteristically, FTY720 phosphate leads to agonist-induced receptor internalization of the S1P_1_ receptor.

Sphingolipids have first been described by the German physician Ludwig Thudichum, then living in London (Great Britain) in the end of the 19th century in his book “A treatise on the chemical constitution of the brain”. The more complex cerebrosides) are required for cell functions such as cell recognition, vesicular transport and lysosomal degradation and their dysregulation can lead to storage diseases with a neurological phenotype (Sandhoff and Christomanou, 1979), for example Tay-Sachs disease, Sandhoff disease or Niemann-Pick disease. The physiological relevance of sphingomyelin and its derivatives as signaling molecules gradually came into focus during the last decades, spurred by the discovery of sphingosine as an inhibitor of protein kinase C (Hannun et al., 1986; Wilson et al., 1986, reviewed by Kolesnick, 1991). Numerous reviews focus on the intricate regulation of the sphingolipid pathway (Huwiler et al., 2000; Hannun and Obeid, 2008; Huwiler and Pfeilschifter, 2008) with ceramides and sphingosine 1-phosphate (S1P) functioning as key signaling molecules (Figure 1). The pathway forms a rheostat and the substrates are readily interconvertible. Ceramide, which is derived either from the membrane lipid sphingomyelin by sphingomyelinases or synthesized de novo can be induced by many cell stressors. It acts on defined intracellular targets (reviewed by Huwiler et al., 2000; Ruvolo, 2003) and in general has proinflammatory and proapototic effects. It is degraded to sphingosine, which can be phosphorylated to S1P by sphingosine kinase (SphK), an enzyme that exists in two isoforms with different subcellular distribution, SphK1 and SphK2. S1P is an antiinflammtory, proproliferative and antiapoptotic signaling molecule. It was first assumed to act as an intracellular second messenger, because its cellular levels increased upon growth factor stimulation of SphK. However, it took years to identify the first intracellular targets of S1P, whereas it was soon discovered that S1P can signal from the extracellular side through a family of G protein-coupled receptors (GPCRs), formerly known as Endothelial Differentiation Genes (EDG; Kluk and Hla, 2002). Previously, they had been orphan receptors without known ligands. In 2002, they were renamed by the IUPHAR into S1P_1–5_ (Chun et al., 2002). Kawahara et al. (2009) identified Spinster 2 (Spns2) as a specific outward transporter for S1P (Figure 2).

Mouse models with genetic deletions of S1P receptors and the use of pharmacological tools have been helpful to shed light on the role of S1P in lymphocyte homeostasis, in the development of the cardiovascular and the central nervous system (CNS), and maintenance of endothelial barriers (reviewed by Brinkmann, 2007). S1P_1–3_ show a practically ubiquitous expression, whereas S1P_4_ expression is mainly detectable on leukocytes but not relevantly in the brain, and S1P_5_ shows a high expression in oligodendrocytes, the main myelinating cells of the brain. The receptors couple to different G proteins (Gαi, Gαq, Gα12/13). From the point-of-view of drug development, the differential receptor expression patterns in different organs, a modulation of receptor expression in disease states, as well as the differential coupling to G proteins, may afford a sufficient degree of specificity to make S1P receptors drugable targets (Figure 2).

Currently, many of the pathophysiologically relevant processes seem to be regulated by the S1P_1_ receptor. Studies of S1P receptor knockout mice showed embryonic lethality for S1P_1_-deficient mice with defects in vasculogenesis and neural tube formation, supporting the important role of this receptor in developmental processes, but not for mice deficient for one of the other four S1P receptors. The S1P_1_ receptor has also been shown to be the relevant receptor to mediate the decisive effect of the vascular S1P gradient on the number of circulating immune cells. S1P concentrations are close to the micromolar range in the bloodstream and concentrations in interstitial fluids are around three orders of magnitude lower. Most prominent are the effects on T lymphocytes: antigen-induced activation of T cells leads to an up-regulation of S1P_1_ and therefore increases the responsiveness to S1P. S1P_1_ regulates T cell trafficking at multiple stages of T cell development as well as their responses, e.g., thymocyte egress into the periphery, egress of mature T cells out of lymph nodes during systemic trafficking and retention of T cells in non-lymphoid tissues. B cells do not require S1P_1_ to leave the bone marrow but, like T cells, they need the receptor to exit secondary lymphoid organs (Matloubian et al., 2004; Ledgerwood et al., 2008; Skon et al., 2013). The egress of other immune cells such as eosinophils (Sugita et al., 2010) and natural killer cells (Walzer et al., 2007) also depends on S1P receptors. In dendritic cells, the functional S1P antagonist fingolimod increased the production of the anti-inflammatory cytokine IL-10 and reduced IL-12 secretion (Durafourt et al., 2011).

The vascular S1P gradient also seems to serve as a “tonic” regulator of endothelial barrier integrity. The intricate regulation of barrier function via cytoskeletal rearrangement with opposing functions of the S1P receptors S1P_1_, S1P_2_ and S1P_3_ signaling via small GTPases of the Rho family and the functional consequences in several disease models have been reviewed by Xiong and Hla (2014; Figure 2).

Besides the S1P receptors, also the SphKs catalyzing S1P formation, SphK1 with cytoplasmic localization and SphK2, the predominant SphK isoform in the brain, with primarily nuclear localization (Igarashi et al., 2003) as well as the S1P degrading enzymes S1P lyase (S1PL; Le Stunff et al., 2002), S1P phosphatases (SPP1 and SPP2), and the lysophospholipid phosphatase 3 (LPP3; Brindley and Pilquil, 2009) have been found to be altered in several neurological diseases and also represent putative targets for drug development (Figure 1).

## Discovery and development of fingolimod/FTY720

**Figure 3:**
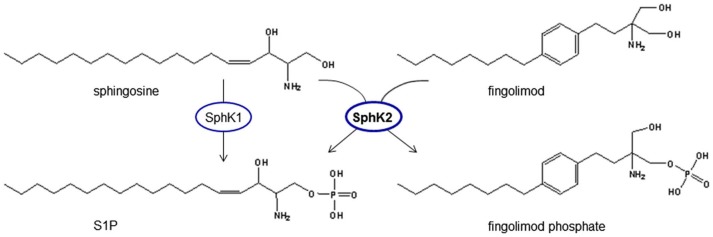
**Phosphorylation of fingolimod generates a structural analog to S1P**. Fingolimod shows high analogy to sphingosine and is phosphorylated by spingosine kinases, mainly SphK2, which is the predominant SphK isoform in the brain. Fingolimod is a prodrug of fingolimod phosphate that can signal via S1P receptors and activate intracellular targets of S1P.

The substance fingolimod (also known as FTY720) has made a very long and interesting journey from traditional asian medicine to a drug tested in large scale international multicenter randomized-controlled placebo-or standard of care-controlled trials proving its efficacy in the treatment of multiple sclerosis (MS) (reviewed by Im, 2003). It evolved from derivatization of ISP-1 that has been isolated from the fungus Isaria sinclairii. This fungus contributes to the fascinating phenomenon of “vegetable wasps and plant worms”, also termed “vegetative wesps” or “winter-insect and summer-plant” according to its Chinese characters, caused by the fungus infecting the living insect host, feeding on it during winter time and growing out of the host insect’s cadaver in summer (Fujita et al., 1994). ISP-1 showed 10-100fold greater immunosuppressant activity than ciclosporin A in experimental models (Fujita et al., 1994) and was modified for less gastric toxicity to fingolimod (Kiuchi et al., 2000). In contrast to all known immunosuppressants known at that time, fingolimod acts via a sequestration of circulating mature lymphocytes to the lymph nodes (Chiba et al., 1998) without major alterations of their immune functions such as cytokine secretion (Yanagawa et al., 1998), and thus seemed to promise the prevention of allograft rejection without a severe general immunosuppression (reviewed by Brinkmann et al., 2000). Inspired by the structural similarity of FTY720 to sphingosine (Figure 3) and the fact that S1P receptors had been identified on lymphocytes, Brinkmann et al. (2002) discovered that fingolimod was phosphorylated by SphKs to fingolimod phosphate and targeted S1P receptors and thereby could prevent experimental autoimmune encephalitis (EAE), the rodent disease model for MS, in rats. Mandala et al. (2002) provided evidence for lymphocyte sequestration in the lymph nodes secondary to S1P receptor activation. Matloubian et al. (2004) established the S1P_1_ receptor to be essential for sensing of the S1P gradient by lymphocytes leading to recirculation and regulation of lymphocyte egress from both thymus and peripheral lymphoid organs. The inhibition of lymphocyte recirculation by fingolimod argues for a functional antagonism of fingolimod phosphate at the S1P_1_ receptor. Indeed, Gräler and Goetzl (2004) described a downregulation of S1P receptors upon treatment with fingolimod and Pham et al. (2008) showed *in vivo* that treatment with fingolimod reduced membrane expression of S1P_1_ on lymphocytes. This proposed functional antagonism of fingolimod at S1P_1_ is in line with the observation that mutant mice that express an internalization-defective S1P_1_ have delayed lymphopenia kinetics in response to fingolimod (Thangada et al., 2010) and that a lymphocytic knock-down of S1P_1_ also inhibits their egress from thymus (Allende et al., 2004).

Fingolimod was advanced into large scale randomized controlled trials to prevent allograft rejection in patients with renal transplantations but finally its further development was abandoned because high doses (2.5 mg/d and 5 mg/d) did not provide sufficient immunosuppression to allow reduction of co-immunosuppressants and was not superior to standard care. There was an increased incidence of macular edema and transient decreases in heart rate (reviewed by Mansoor and Melendez, 2008). Since 10–100–fold lower doses than those required in animal models of organ graft survival had been highly efficient in EAE (Brinkmann et al., 2010), the focus of clinical development shifted from transplant medicine to MS as an autoimmune disease.

**Figure 4:**
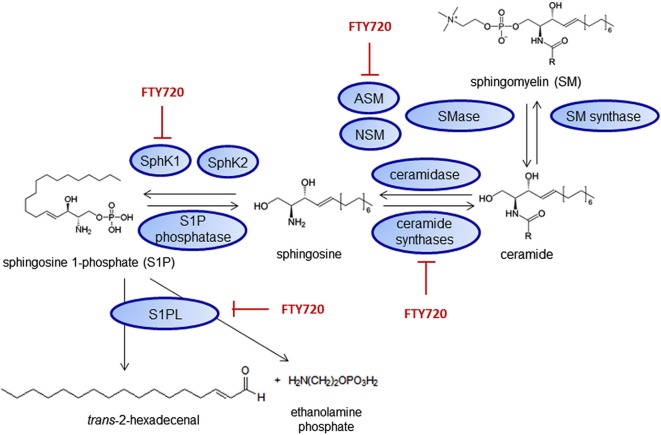
**Fingolimod interacts with sphingolipid metabolizing enzymes**. Inhibitory effects on SphK1 (Lim et al., 2011), S1PL (Bandhuvula et al., 2005), ceramide synthases (Lahiri et al., 2009) and the acid sphingomyelinase (ASM; Dawson and Qin, 2011) have been shown. NSM—neutral sphingomyelinases.

Fingolimod might also have receptor-independent effects on inflammation, especially by binding to intracellular targets of S1P (Hait et al., 2014) or interacting with metabolism and signaling of other lipids. Fingolimod can inhibit both S1P generating as well as degrading enzymes such as SphK1 (Lim et al., 2011), S1PL (Bandhuvula et al., 2005), the ceramide synthases (Lahiri et al., 2009) and the acid sphingomyelinase (ASM; Dawson and Qin, 2011; Figure 4).

Ceramides could also play a role in MS pathophysiology (Qin et al., 2010; Schiffmann et al., 2012) and evidence for an interaction of fingolimod with ceramides has been shown (van Doorn et al., 2012). Fingolimod also appears to inhibit the cannabinoid receptor CB1 (Paugh et al., 2006), which has been shown to have some proinflammatory properties in EAE (Zhang et al., 2009). Additionally, fingolimod can inhibit phospholipase A_2_ (PLA_2_) activity in mast cells and therefore prostaglandin and thromboxane secretion (Payne et al., 2007). This could contribute to the therapeutic effect of fingolimod in MS, as PLA_2_ has been shown to be highly expressed in EAE plaques (Kalyvas and David, 2004) and arachidonic acid is increased in cerebrospinal fluid of MS patients (Dore-Duffy et al., 1991).

## Clinical efficacy of fingolimod in relapsing-remitting multiple sclerosis

MS is an autoimmune disease of the CNS. The primary mechanism is the aberrant formation of autoreactive immune cells directed against CNS antigens. Upon passing the blood-brain barrier (BBB), they meet their antigen and incite inflammatory demyelination leading to subacute neurological symptoms that can remit and, given the chronicity of the disease, relapse. In the early inflammatory phase of the disease, immunomodulation with beta-interferons or glatiramer acetate have been shown to slow disease progression. In later stages of the disease, neurodegenerative processes also play a role.

Fingolimod was approved by the regulatory authorities worldwide as the first oral agent to treat relapsing-remitting multiple sclerosis (RRMS) after the successful completion of two phase III studies in 2010 showed its efficacy. FREEDOMS (Kappos et al., 2010) was a placebo controlled trial and TRANSFORMS (Cohen et al., 2010) even showed the superiority of fingolimod over treatment with intramuscular interferon beta-1a, the standard of care for RRMS. FREEDOMS showed a relative reduction of the relapse rate by 54–60% and a corresponding effect on disability progression (Kappos et al., 2006). TRANSFORMS showed similar relapse rates, although no significant effect on disability. FREEDOMS II, another Phase III placebo controlled trial (Calabresi et al., 2014) initiated by Novartis recently, could reproduce the positive effect of fingolimod on relapse rate. In clinical practice, due to its presumed superiority over interferon beta, fingolimod is one of the first line therapeutic choices for highly active RRMS.

Besides the positive effect on clinical outcome, previous clinical trials (Kappos et al., 2006; Comi et al., 2010) as well as the following phase III studies FREEDOMS, TRANSFORMS and FREEDOMS II have shown effects of fingolimod on magnetic resonance imaging (MRI) outcomes: Gadolinium (Gd) enhanced lesions, the morphological correlates of clinical as well as subclinical relapses, were reduced in the fingolimod treatment group compared to the patients receiving interferon beta-1a or placebo. Gd enhancement indicates BBB leakage, which is one of the initial steps in the pathophysiological cascade resulting in a MS plaque. Furthermore, BBB failure is a hallmark of almost every inflammatory reaction in the CNS. Another secondary endpoint of some of the studies cited above was brain atrophy—which was also reduced in fingolimod treated patients. The reduction of Gd-positive lesions demonstrates a direct or indirect anti-neuroinflammatory effect of fingolimod in patients, whereas the relatively crude parameter brain atrophy might be attributable to a direct neuroprotective mechanism or secondary to a reduced accumulation of MS plaques and secondary neurodegeneration. As expected from the preceding animal studies, peripheral lymphocyte counts of fingolimod-treated patients are significantly reduced as compared to patients receiving placebo and interferon beta-1a (Cohen et al., 2010; Kappos et al., 2010). In contrast to other cytotoxic immunosuppressive drugs resulting in lymphopenia, the effect of fingolimod on the peripheral circulation of subsets of lymphocytes is reversible (Boulton et al., 2012). In line with the available experimental data, other studies showed that fingolimod selectively inhibits the egress of CCR7-positive naive and central memory T lymphocytes from the lymph node of treated patients (Pham et al., 2008). Effector memory cells represent the largest T cell population in the blood of patients receiving fingolimod and are important to maintain immune competence (Masopust et al., 2001). Furthermore, these cells did not lose the capacity to produce interferon-γ (IFNγ; Mehling et al., 2008). On the other hand, the CD4+TH17 subset of lymphocytes, which are trapped by fingolimod is believed to have a more pro-inflammatory role in the pathophysiology of MS (Lock et al., 2002; Tzartos et al., 2008). TH17 cells produce proinflammatory IL-17 and IL-22, transmigrate efficiently across the BBB, disrupt tight junctions, highly express neurotoxic substances, and promote CNS inflammation through additional CD4+ T cell and neutrophil recruitment (Kebir et al., 2007). In fingolimod-treated patients, the remaining CD8+ T cells are less responsive to the chemokine CCL2 (Johnson et al., 2010) and, therefore, probably less likely recruited to sites of antigen-induced inflammation (Carr et al., 1994). Fingolimod also strongly reduces certain sets of B cell counts in the blood of MS patients (Kowarik et al., 2011). The remaining lymphocyte populations appear to be sufficient to protect patients against most infections (Francis et al., 2014). However, there is some evidence for more viral infections, e.g., severe herpes/varizella virus reactivation (Cohen et al., 2010), pointing towards a compromised T cell function. The broad range of immune regulatory functions of fingolimod seen in patient blood samples and those of experimental animals goes far beyond lymphocyte trafficking and has recently been reviewed by Garris et al. (2014).

In summary, the clinical data on the efficacy of fingolimod in RRMS is robust and has been shown in several independent phase III trials. Additional drugs that modulate S1P signaling are in the pipeline, some of them in advanced clinical trials (reviewed by Bigaud et al., 2014). The dose of 0.5 mg/d approved for MS treatment, which is decisively lower than doses used in animal models to treat EAE, also leads to peripheral lymphopenia and other immune cell mediated effects in patients. But the MRI findings that fingolimod has unequivocal effects on BBB disruption and brain atrophy suggests additional direct effects of fingolimod in brain cells.

## Fingolimod in experimental models of multiple sclerosis

Fingolimod was first shown to be effective in EAE in the rat by Brinkmann et al. (2002) at a dose of 0.3 mg/kg/d. Later studies confirmed these results in different models of MS and different species (Webb et al., 2004; Kataoka et al., 2005). The suppression of EAE in mice and rats by fingolimod correlated with reduced numbers of lymphocytes in the blood and the CNS (Brinkmann et al., 2002; Kataoka et al., 2005).

Besides a prophylactic effect, which is comparable to relapse prevention in patients, a therapeutic effect could also be shown (Kataoka et al., 2005). Even a very late-initiated treatment is able to reverse paralysis (Balatoni et al., 2007), downregulates inflammatory genes such as matrix metalloproteinases and reduces BBB leakiness (Foster et al., 2009). This effect is only one of the mechanisms which appears to be independent of the “classical” role of fingolimod in lymphocyte trafficking. In EAE several additional effects on the immune system have been shown: Fingolimod changes CD8+ effector T cell function and inhibits their cytotoxic function by inhibition of cytosolic PLA_2_ and the reduction of IFNγ and Granzyme B expression. Interestingly, this effect appears to be independent of S1P receptor modulation as fingolimod phosphate did not elicit these effects (Ntranos et al., 2014). In some EAE-studies, the effect of fingolimod on CNS physiology was analyzed. The observed clinical benefit was accompanied by improvement of electrophysiological abnormalities (Balatoni et al., 2007), demyelination (Papadopoulos et al., 2010) and synaptic dysfunction (Rossi et al., 2012).

At least some of these outcome parameters might be due to an additional effect of fingolimod directly within the CNS. Fingolimod readily penetrates into the CNS and accumulates in the brain and in the spinal cord (Foster et al., 2007). S1P receptors are expressed in cells of the CNS. Interstingly, short-term fingolimod administration can fail to suppress EAE, albeit producing a rapid and substantial reduction of lymphocyte counts (Foster et al., 2007). Moreover, neurological deficits reappear before lymphocyte numbers normalize if treatment with fingolimod is stopped (Webb et al., 2004). CYM-5442, an S1P_1_-selective fingolimod analog that leads to significant levels of the drug in CNS but not in plasma, also improves the disease course in EAE. There was no persisting lymphopenia in these mice but a cyclical recovery from lymphopenia. Nevertheless, S1P_1_ expression on neurons and astrocytes was reduced, and levels of cytokines in the CNS were suppressed (Gonzalez-Cabrera et al., 2012).

Furthermore, in alternative MS models, which are based on lymphocyte-independent demyelination, an effect of fingolimod could be observed: Fingolimod enhances remyelination following demyelination of organotypic cerebellar slices (Miron et al., 2010) and in the cuprizone model (Kim et al., 2011), although there is some controversy about the latter model (Hu et al., 2011). Another animal model for MS is the delayed-type hypersensitivity model (DTH) in Lewis rats. Again, an effect of fingolimod could be shown independently of lymphocyte infiltration and BBB leakage (Anthony et al., 2014).

So what could be the cellular target of fingolimod within the CNS? There is accumulating evidence for a specific role of astrocytes, a cell type which is supposed to play an important role in MS pathophysiology (Brosnan and Raine, 2013). Strong evidence points to an anti-inflammatory effect of fingolimod on astrocytes *in vitro* (Wu et al., 2013). A particularly illuminating study of Choi et al. (2010) made use of a conditional, cell-specific knock down of the S1P_1_ receptor on all cells of the CNS (nestin-cre), neurons (synapsin-cre) and astrocytes (GFAP-cre). They found that S1P_1_-deficiency on astrocytes led to an attenuation of EAE and that the protective effect of fingolimod in EAE was lost in mice with astrocytic but not with neuronal S1P_1_ deletion (Choi et al., 2010). Finally, therapeutic administration of fingolimod to EAE mice has specific effects on astrocyte activation and nitric oxide production (Colombo et al., 2014).

Oligodendrocytes also express S1P receptors, especially the S1P_5_ receptor, which shows a relatively restricted expression pattern. Interestingly, genetically modified mice with S1P_5_ deficiency do not show defects in myelination (Brinkmann, 2007). At least *in vitro* and in slice cultures direct effects of fingolimod on myelination could be shown (Miron et al., 2008, 2010). A possible role for microglia in the therapeutic effect of fingolimod can be derived from studies of so called microvesicles. They were shown to be significantly reduced in EAE mice treated with fingolimod and are increased in cerebrospinal fluid of MS patients (Verderio et al., 2012). Furthermore, fingolimod treatment leads to a reduced microglial production of pro-inflammatory cytokines such as tumor necrosis factor α (TNFα), interleukin 1β (IL-1β), and interleukin 6 (IL-6; Noda et al., 2013).

An analysis of fingolimod’s effects in EAE clearly points towards several contributing mechanisms. Beyond the effect on lymphocyte trafficking, a direct immunmodulatory effect as well as direct effects of fingolimod in the CNS seem to be involved. As new, more receptor-specific agents will enter the scene in the future, it is possible that we will realize that fingolimod as a rather “dirty” drug acting on multiple targets may even prove to be superior to receptor-specific agents.

## Stroke

Stroke is an acute brain attack caused by the sudden thromboembolic occlusion of a brain vessel with the consequence of cerebral ischemia, the more frequent cause of stroke, or by a rupture of a brain artery causing a hemorrhage into the brain parenchyma. These different stroke entities show the same clinical presentation consisting of a focal neurological deficit such as hemiparesis or aphasia of sudden onset.

### Cerebral ischemia

The embolic blockade of a brain vessel leads to hypoperfusion of the dependent vascular territory in the brain with an almost immediate loss of brain function. However, we know from experimental studies (Astrup et al., 1981) and clinical imaging (Thijs et al., 2002) that the affected brain tissue remains viable for a few hours with the infarct gradually expanding from the core to the periphery of the hypoperfused territory. This is the pathophysiologic correlate of a therapeutic time window for vessel recanalization with thrombolytics or catheter-based interventions. The only approved stroke therapy to date is thrombolysis with recombinant human tissue-type plasminogen activator (t-PA), which is safe and effective within 4.5 h after symptom onset (Lees et al., 2010). The pathophysiological cascades taking place in the ischemic brain are well characterized and can be roughly summarized by the three interleaving phenomena of excitotoxicity, inflammation and apoptosis (Dirnagl et al., 1999).

In recent years, experimental stroke research has produced strong evidence that cellular immunity and especially T cells play a decisive role in the fate of brain tissue following cerebral ischemia. Different experimental strategies aiming at a reduction of lymphocyte counts such as the use of genetic models (Hurn et al., 2007; Kleinschnitz et al., 2010); splenectomy prior to stroke (Ajmo et al., 2009), immunosuppressive drugs (Sharkey et al., 1996) or the induction of lymphocytic tolerance (Becker et al., 1997) have shown therapeutic efficacy in experimental stroke models. Therefore, to test the potent immunomodulator fingolimod with its specific lymphocyte-directed immunomodulation in experimental stroke models was a straightforward approach.

Meanwhile, over 10 experimental studies have shown therapeutic efficacy of fingolimod in stroke models in mice and rats (Czech et al., 2009; Shichita et al., 2009; Hasegawa et al., 2010; Pfeilschifter et al., 2011a,b; Wei et al., 2011; Kraft et al., 2013) with doses from 0.25 to 1 mg/kg applied systemically, leading to a robust lymphocytopenia in rodents (Czech et al., 2009). Reproducible therapeutic effects on lesion size and functional outcome were accompanied by a reduction in pro-apoptotic processes in the infarcted brain areas such as reduced nuclear translocation of apoptosis inducing factor (AIF; Czech et al., 2009), less caspase-3 cleavage and TUNEL positive neurons (Hasegawa et al., 2010), and an activation of pro-survival pathways such as extracellular signal-regulated kinase (ERK1/2), protein kinase B/Akt kinase (PKB/Akt) and Bcl-2 upregulation (Hasegawa et al., 2010; Wei et al., 2011). Cell culture experiments did not show relevant neuroprotection by fingolimod in neuronal cells subjected to “ischemia in the dish” stimuli such as glutamate, H_2_O_2_ or hypoxia (Wei et al., 2011; Kraft et al., 2013) but a reduced inflammatory activation of microvascular brain endothelial cells (Wei et al., 2011). Most topical, Kraft et al. (2013) found a reduction of microvascular thromboses in the periinfarct area after fingolimod treatment, a phenomenon that has been observed also previously by the group secondary to several T cell-directed therapies (Kleinschnitz et al., 2013). Since these findings argue for a role of lymphocytes to boost microvascular thrombus formation in cerebral ischemia, they coined the term thromboinflammation. Of note, there was also one study that reported no therapeutic effect on lesion size and outcome in two different stroke models despite efficient systemic lymphocyte depletion, and decreased cerebral lymphocyte infiltration (Liesz et al., 2011). Similar findings were reported in a recent study on traumatic brain injury (TBI) by Mencl et al. (2014), who assessed the effect of FTY720 in two different experimental models of TBI mimicking focal and diffuse brain injury. FTY720 applied directly prior to the induction of the brain lesion did not reduce lesion size or improve functional neurological outcome neither early or 1 week after the induction of the brain lesion, even though it led to a reduced infiltration of neutrophils and macrophages and/or a reduced microglial activation. TBI, however, is a clearly different entity of brain injury than ischemic stroke. The role of circulating immune cells in lesion development after TBI is less unequivocal and there is most probably not as much microvascular endothelial activation as in ischemic stroke.

Concerning the signaling pathway mediating the protective effect of fingolimod, Hasegawa et al. (2010) demonstrated that this therapeutic effect could also be elicited by the S1P_1_-selective agonist SEW2871 and abrogated by the S1P_1_/S1P_3_-selective competitive antagonist VPC23019, argueing for an S1P_1_-mediated effect. This assumption was supported by findings of Pfeilschifter et al. (2011b) showing that the neuroprotective effect of fingolimod is lost in SphK2 deficient mice, which cannot efficiently phosphorylate and thus activate fingolimod, arguing against a direct effect of non-phosphorylated fingolimod. Hasegawa et al. (2013) examined the expression of the SphK and the S1P_1_ receptor in the ischemic area with a focus on neurons 6 h and 24 h after stroke by immunohistochemistry, and found a significantly decreased expression of S1P_1_, SphK_1_, and SphK_2_ starting at 6 h and significant at 24 h after MCAO. Labeling of S1P_1_ and both SphKs was reduced in the infarct cortex but remained present in the periinfarct cortex, allowing fingolimod to be phosphorylated and act via inside-out signaling on the S1P_1_ receptor.

In analogy to the findings of Choi et al. (2010) in the EAE model, it could be possible that the protective effect of fingolimod only partially depends on the peripheral immunomodulation and also relies on direct effects in the CNS. To weigh the effect of lymphocyte depletion against other putative mechanisms, Kraft et al. (2013) applied fingolimod to recombination activating gene-1 (Rag1)-deficient mice that are devoid of T and B lymphocytes. While these mice *per se* developed smaller infarcts than wild type mice, they were not further protected by fingolimod, showing that the neuroprotective effects of fingolimod largely depends on immunomodulation.

Stroke-associated infections, especially pneumonia, are the most important factor of mortality in the acute phase. They are promoted by a stroke-associated immunodepression (Meisel et al., 2005) and will have to be considered prior to any clinical application of fingolimod in the context of stroke. In an experimental stroke model, Pfeilschifter et al. (2011b) did not find an increase of bacterial colonalization in the lungs of fingolimod-treated animals in comparison to control animals 24 h after stroke. Beyond the acute phase, the fate of stroke survivors is mainly determined by the success of rehabilitative stroke care aiming at functional recovery. Brunkhorst et al. (2013) showed that fingolimod applied from day 3 to day 7 (1 mg/kg b.i.d.) after a photothrombotic lesion directed to the motor cortex of mice relevantly ameliorated functional impairment over an observation period of 31 days, reduced astroglial scarring and increased the size of post-synaptic densities. In this model, fingolimod increased the expression of the trophic factor vascular endothelial growth factor α (VEGFα) but not of brain-derived neurotrophic factor (BDNF), which has been shown to increase secondary to fingolimod treatment in other models of neurodegenerative diseases.

Another event of importance in the context of stroke, but also intensive care medicine and transplant surgery, which has been shown to be regulated by S1P and fingolimod is preconditioning. Following the principle of “what does not kill you makes you stronger”, preconditioning is a process that uses a sublethal noxious stimulus to induce or increase tolerance towards a second noxious stimulus. In stroke research, well established preconditioning stimuli include hypoxic preconditioning (HP), transient ischemia, inhalational anesthetics such as isoflurane or inflammatory agents such as bacterial lipopolysaccharide (LPS). HP is based on a hypoxia-induced stabilization of hypoxia-inducible factor (HIF) that is continuously degraded by an oxygen-sensing degradative pathway under normoxic conditions. Stabilized by hypoxia, HIF, which exists in three isoforms, binds to hypoxia-responsive elements (HRE) of hypoxia-regulated genes to promote their transcription. Besides hypoxia and ischemia, also non-hypoxic stimuli such as isoflurane (Sun et al., 2013) or LPS (He et al., 2014) can stabilize HIF. SphK2, but not SphK1 has been shown to be upregulated by HP and isoflurane in the brain (Wacker et al., 2009; Yung et al., 2012) in a HIF-dependent manner (Wacker et al., 2012) and pharmacological inhibition (Wacker et al., 2009) and genetic deletion of SphK2 (Wacker et al., 2012; Yung et al., 2012) were shown to abrogate the protective effects of preconditioning in experimental stroke. There are conflicting findings whether genetic deletion of SphK2, the predominant SphK isoform in the brain (Blondeau et al., 2007) *per se* exacerbates ischemic damage in stroke or not (Pfeilschifter et al., 2011b; Yung et al., 2012). In other cell systems, HIF-mediated upregulation has also been shown for SphK1 (Ader et al., 2008; Schwalm et al., 2008) and both isoforms contain HREs in their respective promotor regions. Wacker et al. (2009, 2012) showed that fingolimod given 48 h prior to stroke also reduced lesion size and had a strong synergistic effect in conjuction with HP. Therefore, they concluded that fingolimod is a preconditioning agent. While the protective effect after a remote pretreatment 48 h prior to the insult could be explained by the long-lasting lymphocyte depletion after a single dose of FTY720 (Chiba et al., 1998) and the accumulation of fingolimod in the brain (Foster et al., 2007), the finding that coadministration of R59949, which inhibits HIF accumulation was able to abolish the protective effect of remote fingolimod pretreatment (Wacker et al., 2012) clearly suggests that HIF-mediated gene regulation is downstream of fingolimod. The finding of Yung et al. (2012) that HIF-1α stabilization following isoflurane preconditioning is lost in SphK2-deficient mice also supports a regulation of HIF-mediated signaling by S1P. In synthesis, these findings suggest that in the context of preconditioning and stroke, the signaling between SphK2 and its products fingolimod phosphate or S1P is bidirectional with an induction of SphK2 following preconditioning stimuli and a lost efficacy of HIF-stabilizing factors in SphK2-deficient mice on the one hand and a HIF-dependency of the preconditioning effect of fingolimod on the other hand. The preconditioning effect of HP, the HIF stabilizer cobalt chloride, and of fingolimod was abolished by pretreatment with the S1P_1_-specific antagonist W146, supporting an auto- and paracrine propagation of preconditioning by the S1P_1_ receptor. Functionally, SphK2 deficiency not only abrogates the neuroprotection by HP but also BBB preserving effects of HP by an alteration in the expression of adhesion molecules in homogenated cortical brain tissue from SphK2-deficient mice analyzed 48 h after the hypoxic stimulus in comparison to WT mice (Wacker et al., 2012).

### Thrombolysis with tissue plasminogen activator (t-PA)

The vascular S1P gradient with high concentrations in the bloodstream and low concentrations in the interstitial fluids is generally viewed as a tonic barrier preserving mechanism for endothelial barriers (reviewed by Xiong and Hla, 2014). Several reports of the efficacy of S1P or fingolimod in experimental states of vascular leakage such as acute lung injury (Peng et al., 2004) or anaphylaxis (Camerer et al., 2009) have sparked interest on a putative therapeutic effect of fingolimod on BBB disruption after stroke that contributes to potentially fatal brain edema. So far, the data from experimental stroke models on this question are not unequivocal. Cerebral ischemia induces BBB disruption and the thrombolytic t-PA has been shown to aggravate this process (Latour et al., 2004) which accelerates with time from symptom onset, representing the underlying cause of an increased bleeding risk associated with late t-PA treatment, the main obstacle to a more widespread use of this stroke therapy. In a model of moderate size thromboembolic stroke, a faithful model for recanalization by t-PA treatment, fingolimod reduced infarct growth and BBB disruption secondary to late application of t-PA (Campos et al., 2013). By contrast, in a model of large hemispheric strokes and t-PA treatment, fingolimod did not show a beneficial effect (Cai et al., 2013). These contradictory findings can in part be reconciled by the possibility that this massive infarction might have led to an overly rapid loss of SphK and S1P receptors in the infarct and periinfarct region explaining a non-response to fingolimod treatment. Concerning possible future clinical trials of fingolimod in stroke, this would argue against the inclusion of patients with severe stroke symptoms and massive infarctions on brain imaging.

### Intracerebral hemorrhage

Intracerebral hemorrhage (ICH) is often denoted as “the most untreatable cause of stroke” because there is no causal treatment and it is charged with a high mortality. Noteworthy, Asian populations display higher rates of ICH, with ICH representing over 30% of all first ever strokes in China in a meta-analysis of population-based studies (Tsai et al., 2013). Fingolimod has shown therapeutic efficacy in experimental models of ICH (Rolland et al., 2011, 2013; Lu et al., 2014) with a reduced infiltration of CD3+ lymphocytes and reduced endothelial ICAM-1 expression. Rolland et al. (2013) conducted a 10 week follow-up of their experimental animals after ICH induction and a single injection or a 3-day course of 1 mg/kg FTY720 and auspiciously found an improved outcome in neurocognitive scores and brain atrophy with a stronger effect in the animals treated for 3 days. These findings support the observations of a proregenerative effect of FTY720 by Brunkhorst et al. (2013). Lu et al. (2014) described a reduction of apoptotic cell death both in the center and at the edge of the hematoma but no alteration of in the numbers of CD68+ monocytes/macrophages and resident microglia and also reported positive long-term results concerning neurological function and brain atrophy after 2 weeks.

Recently, a first clinical trial was conducted in China, which recruited 23 patients with deep primary ICH. They were randomized to a treatment with placebo or 0.5 mg/d FTY720 (Gilenya®) orally for 3 consecutive days (Fu et al., 2014). This very small clinical trial did not detect differences in adverse events such as increased susceptibility to infections. The group reported an improvement of magnetic resonance imaging-based measurements of perihematomal edema volume, but the patient number seems too small to draw valid conclusions about efficacy yet.

## Neurodegenerative diseases

### Alzheimer’s disease

Alzheimer’s disease (AD) is the most common age-related neurodegenerative disorder and shows a rising prevalence along with global trends towards increasing life spans. AD leads to a cognitive decline that is attributable to neuronal loss predominantly affecting higher cortical functions (visuospatial orientation, memory, planning and execution of activities, language and arithmetics) whereas cognitive working tempo and drive seem unaltered. Patients are often able to maintain an unremarkable “façade” even in later stages of the disease when brain atrophy, predominantly of the hippocampus and the temporal lobe can be seen in clinical brain imaging. Currently, there is no causal treatment of this devastating disease. While the disease-triggering mechanisms are yet unknown, the neuropathological hallmarks of AD consist of the deposition of amyloid-β (Aβ) plaques and the formation of neurofibrillary tangles consisting of hyperphosphorylated tau protein (Braak and Braak, 1991, 1995). Following an ascending course from the entorhinal region to the neocortex, these changes are accompanied by a loss of cortical synapses, which correlates with cognitive decline (DeKosky and Scheff, 1990). Inflammatory processes such as activation of microglia and astrocytes as well as production of inflammatory cytokines occur within and around amyloid plaques (Lukiw and Bazan, 2006). Recently, autopsy studies on brains of AD patients but also data from experimental disease models of AD have provided evidence that alterations of the sphingolipid linked to the pathological mechanisms of AD and that S1P receptor modulation by fingolimod represents a promising therapeutic approach reducing both the production (Takasugi et al., 2013) and the neurotoxicity (Asle-Rousta et al., 2013; Doi et al., 2013; Hemmati et al., 2013; Fukumoto et al., 2014) of Aβ peptide. Aβ is produced from Aβ precursor protein (APP) in the amyloidogenic pathway through sequential cleavage by two aspartate proteases, β- and γ-secretases (Zheng and Koo, 2011). Several studies have implicated cholesterol and sphingolipid-rich membrane microdomains, termed lipid rafts, in the amyloidogenic processing of APP, and the activities of the secretases are influenced by the composition of these lipid rafts (Kalvodova et al., 2005; Osenkowski et al., 2008; Holmes et al., 2012).

There is ample evidence that the sphingolipid metabolism is altered in AD brains, leading to an accumulation of proapoptotic and proinflammatory ceramides (Han et al., 2001). The expression of the ceramide-generating acid and neutral sphingomyelinases (NSM) has been shown to be upregulated in brains of patients suffering from AD (Filippov et al., 2012) and elevated ceramide levels are linked to an inhibition of glycolysis (Arboleda et al., 2007) and the induction of nitric oxide synthase and oxidative stress (Cutler et al., 2004). Cutler et al. showed elevations of cholesterol levels and ceramide species (especially C18 and C24) that gradually increased in correlation to disease severity in brain samples from AD patients and over time in a murine model of AD. The accumulation of ceramide species or cholesterol in cultured hippocampal neurons could be reversed by anti-oxidative treatment with α-tocopherol. Mechanistically, this may be attributed to an activation of the neutral sphingomyelinase by Aβ, as Jana and Pahan (2010) had shown previously that Aβ induces neutral sphingomyelinase in an NADPH-dependent pathway leading to ceramide accumulation that has a propapototic effect on cultured neurons. Elevated ceramide levels stabilize the Aβ-cleaving enzyme BACE-1 (Puglielli et al., 2003) whereas a reduction of ceramide levels in turn leads to a reduced secretion of APP and Aβ in human neuroblastoma cells (Tamboli et al., 2005), indicating a vicious cycle involving Aβ production, cellular stress and ceramide accumulation. The clinical significance of increased ceramide levels in the pathophysiology of AD is supported by studies on patient serum and CSF samples that indentified ceramide species as potential biomarkers of AD (Mielke et al., 2014) and HIV-associated dementia (Bandaru et al., 2007).

Less is known about the role of S1P, the functional opponent of ceramide, in the development of AD. An analysis of post-mortem brain tissues of 9 individuals with a clinical and histopathological diagnosis of AD and 6 age-matched control individuals by He et al. (2010) revealed a pattern of elevated ASM and acid ceramidase (AC) expression in AD, along with a reduction in sphingomyelin and elevation of ceramide. Sphingosine levels were higher in the AD brains, but levels of the downstream lipid mediator S1P were reduced. This finding was corroborated by a very recent study of Couttas et al. (2014) reporting on sphingosine/S1P levels and SphK activity in post-mortem brain samples from 34 individuals. Not all of the patients had received a clinical diagnosis of AD and the cohort was composed of carriers of the protective apolipoprotein (APO) ɛ2 genotype as well as the risk-bearing APO ɛ4 genotype. In this cohort, the S1P to sphingosine ratio was higher in hippocampal regions of Apo ɛ2 carriers and S1P levels declined in a regiospecific manner most strongly in brain regions with the most advanced histopathological changes. The activity of SphK1 and SphK2, which generate S1P from sphingosine, also decreased with higher histopathological disease scores. Besides a loss of SphK activity, lower S1P levels in AD patients could also be explained by increased activities of the S1P degrading enzymes S1PL and S1PP. Indeed, S1PP1 has been shown to be markedly up-regulated in AD brains using microarray technology (Katsel et al., 2007).

Seemingly in contrast to these findings, Takasugi et al. (2011) described that the activity of β-site APP cleaving enzyme-1 (BACE1), the major β secretase and rate-limiting enzyme for amyloid-β peptide (Aβ) production, is positively modulated by S1P in mouse neurons. They found that β-cleavage of APP was inhibited by genetic knockdown of the S1P-generating SphKs in cultured neuronal cells, an effect that was more pronounced when SphK2 was targeted, or by pharmacological inhibition with the SphK-selective inhibitor SKII. Congruently, increasing S1P levels via inhibition of S1PL or the S1PP1 enhanced Aβ secretion. Modifications of extracellular levels of the poorly permeating lipid mediator S1P failed to modulate BACE1 activity and BACE1 was specifically pulled down to matrices carrying S1P. Therefore Takasugi et al. (2011) concluded that the S1P-BACE1 interaction was of a direct intracellular nature and not mediated via membrane-bound S1P receptors. Translating these findings into a mouse model of AD, they found a protective effect of the SphK inhibitor SKII against Aβ secretion. Since SphK2 is the predominant isoform of the two S1P synthesizing enzymes in the brain (Blondeau et al., 2007) they analyzed SphK2 expression levels and activity in brain specimens from AD patients in comparison to non-demented individuals. While the overall SphK2 protein levels were decreased in the brains of individuals suffering from dementia, which was explained by progressive neuronal loss and the predominantly neuronal expression of SphK2 in the brain (Blondeau et al., 2007) the relative *in vitro* activitiy of SphK2 normalized to SphK2 protein levels was significantly increased in AD brains (Takasugi et al., 2011). Due to the normalization of total SphK2 protein levels, these findings do not necessarily stand in contrast to the above-mentioned observations of Couttas et al. (2014) who found a decreased SphK activity of both isoenzymes in post-mortem brain specimens from individuals with histopathological features of AD.

A very recent report from Karaca et al. (2014) on the influence of S1P levels in models of S1PL deficiency on APP metabolism sustained detrimental effects of elevated intracellular S1P levels concerning Aβ accumulation. This group found that inhibition of SphK2 reduced the accumulation of amyloidogenic C-terminal fragments of APP whereas genetic deletion of S1PL or downregulation by siRNA, which both were effective in raising intracellular S1P levels, increased their accumulation. This effect could be mimicked by extracellular addition of the easily cell-permeating sphingosine but not by extracellular addition of the polar mediator S1P. Karaca et al. found that the processing of APP C-terminal fragments to Aβ1-40 and Aβ1-42 was reduced due to an impaired γ-secretase activity in S1PL-deficient cells and APP C-terminal fragments accumulated in the lysosomal compartments, along with a deregulated expression of other lysosomal proteins and an impairment of the final stage of autophagy. Interestingly, these pathophysiological processes could be alleviated by a mobilization of intracellular calcium from the endoplasmic reticulum, a process that has been shown to be impaired in a model for the lysosomal storage disease Nieman Pick type C (Lloyd-Evans et al., 2008).

Concerning a putative therapeutic effect of fingolimod on AD progression in experimental models of established disease, there are several reports presenting promising evidence. In a model of intrahippocampal injection of Aβ1-42 in rats, a 2 week course of fingolimod treatment (1 mg/kg daily i.p.) ameliorated spatial learning and memory in the Morris water maze test at the end of the 2 week course and reduced neuronal damage and caspase-3 activation in the hippocampus (Asle-Rousta et al., 2013). Of note, fingolimod did not enhance learning and memory in control animals who received saline injections into the hippocampus. Fingolimod showed equal efficacy as the NMDA receptor antagonist memantine, an approved drug to treat AD symptoms in the same disease model (Hemmati et al., 2013) and both were able to partly reverse changes of the gene expression pattern induced by Aβ injection. So far, the mechanism underlying these therapeutic effects is not yet clear. It is possible that the systemic immunomodulation dampens the inflammatory reaction to Aβ deposition. Specifically, fingolimod may interfere with the migration of phagocytes in the brain since it has been shown to reduce Aβ-triggered whole blood cell migration along an Aβ gradient in a Boyden chamber assay in a dose-dependent manner (Kaneider et al., 2004).

Takasugi et al. (2013) analyzed the effects of fingolimod and the S1P_1_-specific receptor agonist KRP203 on Aβ production in cultured neuronal cells and brain Aβ levels in a murine AD model (Takasugi et al., 2013). In cell culture, they found a dose-dependent reduction of Aβ production by both compounds. Contrary to S1P, fingolimod and KRP203 affected the activity of γ- but not β-secretases. Phosphorylation of both compounds by SphK2 was a prerequisite for their activity as demonstrated by pharmacological inhibition and genetic knockdown of this enzyme. The effects of fingolimod and KRP203 seem to be receptor-independent since Aβ production was not affected by the S1P_1_ agonist SEW2871 or the S1P_1_ antagonist W123, and neither W123 nor the Gαi protein inhibitor suramin altered the inhibition of Aβ production by fingolimod. These findings point towards intracellular effects of fingolimod requiring prior phosphorylation by SphK2, and this hypothesis was further corroborated by the finding that extracellular addition of the polar fingolimod phosphate, which poorly permeates cell membranes, also did not effect Aβ production. Takasugi et al. translated these findings into an animal model of transgenic mice overexpressing human APP and could show opposite effects of a short-course treatment with fingolimod (0.5 mg/kg for 6 days) on the production of Aβ1-40 (decreased) and Aβ1-42 (increased), thus showing that fingolimod affects Aβ levels also *in vivo* (Takasugi et al., 2013).

Furthermore, direct effects of fingolimod on brain cells have been shown, such as a dose-dependent induction of BDNF production in cultured neurons on mRNA and protein levels that inhibited Aβ-induced neurotoxicity in a dose-dependent fashion (Doi et al., 2013). The trophic effects of BDNF were shown to be mediated via the tropomyosin receptor kinase B (TrkB) involving the main downstream pathway ERK1/2 phosphorylation. BDNF was also induced by fingolimod in a murine AD as shown by a BDNF ELISA from mouse brain homogenates (Fukumoto et al., 2014). The translational relevance of these findings is further sustained by the fact that BDNF expression levels are lower in patients with AD.

### Animal models of learning and memory

There is increasing evidence that S1P signaling also plays a physiological role in learning processes. S1P has been shown to increase glutamate release from presynaptic terminals in the CA3 pyramidal neurons of the hippocampus in an S1P_3_ receptor-dependent manner (Kanno et al., 2010). S1P induced the translocation of S1P_3_ receptors to presynaptic mossy fiber terminals in the CA3 region and long term potentiation depending on the mossy fiber—CA3 pyramidal neuron interaction was inhibited by a SphK inhibitor, an effect that could be reversed by addition of S1P. Again, the positive effect of S1P on long-term potentiation (LTP) could be inhibited by S1P_3_ receptor antagonism. SphK1 is highly expressed in mossy fibers of the hippocampus and LTP was significantly impaired in SphK1 knockout mice, showing that SphK1 is required for hippocampal S1P generation. Congruently, mice with genetic deletion of SphK1 showed an impairment of spatial learning and memomory in the Morris water maze. Interestingly, somewhat opposing findings have recently been described by Hait et al. (2014), see below.

Topical work of the last few years has established a role for coagulation factors such as thrombin, plasmin and activated protein C (aPC) and protease-activated receptors (PAR) in hippocampal learning processes (Maggio et al., 2008; Mannaioni et al., 2008; Yuan et al., 2009). Interestingly, Maggio et al. (2014) characterized aPC as a metaplastic molecule that enhances LTP upon delivery of a subthreshold stimulation. They propose a model in which upon binding to the endothelial protein C receptor, which is also expressed on astrocytes, aPC activates PAR1, which triggers the SphK-dependent production of S1P. In turn, S1P binds to the S1P_1_ receptor and stimulates intracellular Ca^2+^ stores, ultimately leading to enhanced LTP.

Very recently, Hait et al. (2014) showed that fingolimod affects learning and memory function by epigenetic regulation of gene expression. After SphK2 dependent phosphorylation, fingolimod phosphate binds to class 1 histone deacetylases (HDAC), some of the few defined targets of intracellular S1P signaling (Hait et al., 2009). Primary hippocampal neurons as well as a neuroblastoma cell line showed a robust, predominantly nuclear expression and activity of SphK2, showing abundant fingolimod phosphorylation within hours of fingolimod treatment. Most of the fingolimod phosphate remained intracellularly, and interfering with SphK2 expression crucially regulated fingolimod phosphate levels. Interestingly, treatment with fingolimod reduced nuclear S1P levels, probably by competition for phosphorylation by SphK2. As shown for S1P in non-neuronal systems (Hait et al., 2009), fingolimod treatment led to histone acetylation, and the negative modulation of this effect by SphK downregulation in a cell membrane-free system of highly purified nuclei as well as the inefficacy of extracellularly-added S1P or fingolimod phosphate showed that this is a purely intracellular inhibitory effect of phosphorylated fingolimod on HDAC activity while the activity of histone acetyltransferases, which have opposing effects, is not influenced by S1P or fingolimod phosphate.

To test the physiological relevance of these observations and following reports on the role of epigenetic regulation, especially histone acetylation in memory and learning processes (Fischer et al., 2010), Hait et al. (2014) chose an experimental model of contextual fear extinction to evaluate the effects of FTY720 on learning and memory *in vivo*. To exclude well-established complex effects of the immune system on cognitive functions (Kipnis et al., 2004; Brynskikh et al., 2008), T and B cell deficient severe combined immunodeficiency (SCID) mice were used. The contextual fear extinction test evaluates freezing as an expression of acquired fear secondary to an adversive stimulus, and fear extinction upon re-exposure to the same environment without this stimulus and hence is a model for how the individual reduces fear-related reactions to a no longer dangerous stimulus, a mechanism that is impaired in anxiety disorders. Here, fingolimod, which accumulated in the hippocampus, did not change fear acquisition and fear extinction, but alleviated delayed extinction deficits in comparison to saline-treated SCID mice. By contrast, performance in paradigms of tone-dependent fear conditioning that is acquired independently of the hippocampus, exploratory behavior, basal anxiety-like behavior and spatial learning were not influenced by fingolimod treatment in SCID mice. The amelioration of contextual fear extinction in the foot-shock model was accompanied by increased histone acetylation in the hippocampus and alterations in gene expression of genes linked to synaptic plasticity and learning. In electrophysiological recordings from hippocampal slices, Hait et al. (2014) could show that like other HDAC inhibitors, fingolimod increased LTP as a marker of synaptic plasticity. To confirm the role of SphK2 in hippocampal learning processes and especially SphK2 dependent phosphorylation of fingolimod, they used SphK2 deficient mice, which spontaneously showed impaired visuospatial memory in the Morris water maze and decreased contextual fear extinction. Consistent with the hypothesis that nuclear phosphorylation of fingolimod by SphK2 is a prerequisite of HDAC inhibition, fingolimod failed to rescue fear extinction in this model.

### Huntington disease

Huntington disease (HD) is an inherited neurodegenerative brain disease with autosomal-dominant inheritance. It is characterized by mutations of the huntingtin gene with extended CAG trinucleotide repeats encoding a dysfunctional mutant huntingtin protein (mHtt). mHtt forms aggregates that are cytotoxic and interfere with many physiological cell functions, such as the expression of neurotrophins, e.g., BDNF (Zuccato et al., 2001). mHtt accumulation has also been shown to influence lipid metabolism (Maglione et al., 2010) and intraventricular infusion of ganglioside GM1 induced phosphorylation and detoxification of mutant huntingtin (Di Pardo et al., 2012). Clinically, this disease which predominantly affects the striatum and the cortex manifests as a hyperkinetic movement disorder with unvoluntary dance-like movements accompanied by a cognitive and psycho-social decline. Symptoms usually begin around the age of 40 and characteristically show an “anticipation” (earlier onset and heavier symptoms from generation to generation) that is linked to an increasing extension of the CAG repeats. Di Pardo et al. (2014) assessed a potential therapeutic effect of fingolimod in transgenic mice expressing exon 1 of the human HD gene carrying a CAG repeat (line R6/2). These mice develop a progressive neurological phenotype with motor symptoms resembling those seen in HD (Carter et al., 1999). Daily fingolimod injections over several weeks, starting in the subtle phase of HD symptoms, significantly ameliorated motor signs of HD and prevented weight loss usually associated with this disease but had no effect on motor tests in WT mice. This was accompanied by activation of the prosurvival pathways of PKB/Akt and ERK1/2 in the striatum. Fingolimod-treated R6/2 mice showed a reduction of brain atrophy and a dramatic reduction of mHtt aggregates in comparison to untreated mice. Fingolimod was able to partially prevent the reduction in corpus callosum thickness that is observed in R6/2 mice compared to WT mice, a hint towards brain-specific and disease-modifying properties of this drug, which were also supported by increases in DARPP-32, a specific marker of medium spiny neurons and myelin-associated glycoprotein (MAG), a marker of myelin and white matter integrity in R6/2 mice after fingolimod treatment. DiPardo et al. also found a significant increase of cortical BDNF mRNA levels both in R6/2 and WT mice treated with FTY720 and the treatment improved cortical neuronal activity. Interestingly, FTY720-treated mice showed a less pronounced loss of GM1 gangliosides in the striatum, so one putative protective mechanism of FTY720 is preventing the disturbance of the lipid balance.

### Rett disease

Rett disease is a rare congenital neurodegenerative disease with postnatal onset, usually at the age of 6–18 months, linked to a mutation in the MeCP2 gene located on the x chromosome. Typical for this disease is a relatively normal appearance of infants at birth followed by a developmental regression with severe motor and cognitive deficits. So far, there is no causal treatment. Experiments with MeCP2 -deficient mice have shown an improvement of symptoms by the administration of a small molecule agonist to the neurotrophin receptor TrkB that is activated by BDNF (Schmid et al., 2012). The fact that BDNF levels in MeCP2 mice did not increase in the first weeks after birth as in WT mice (Kolbeck et al., 1999; Chang et al., 2006) as well as a more severe phenotype of MeCP2 null mice crossed with BDNF-deficient mice or an ameliorated phenotype if MeCP2 null mice were crossed with mice overexpressing BDNF (Chang et al., 2006) pointed towards an influence of BDNF on the course of Rett disease. Furthermore, mice lacking MeCP2 and BDNF in neurons show behavioral similarities (Rauskolb et al., 2010). This makes MeCP2-deficient mice a useful tool to study functional consequences of therapies that raise BDNF levels in the brain. Due to its excellent brain permeability, its confirmed neuroprotective properties in models of cerebral ischemia and the fact that it activates ERK1/2 in neurons–a pathway that is activated by BDNF via the TrkB receptor–prompted Deogracias et al. (2012) to investigate the effects of fingolimod on BDNF levels and disease severity in MeCP2 knockout mice. They found that fingolimod phosphate increased BDNF mRNA and protein in a time- and dose-dependent manner in cultured neuronal cells that were shown to express the S1P receptors S1P_1–3_. The phosphorylation of ERK1/2 and PKB/Akt that occurred in parallel could be shown to be S1P_1_ dependent by use of specific agonists and antagonists whereas it was not further clarified whether BDNF induction was an S1P receptor-mediated or direct intracellular effect. In organotypic cortical cultures, Deogracias et al. (2012) showed that addition of fingolimod phosphate increases network activity and by addition of a monoclonal BDNF-blocking antibody could prove that BDNF is in part responsible for this increased activity. This neutralizing BDNF antibody also reversed the antiapoptotic effect of fingolimod phosphate protecting neurons from NMDA-induced death. *In vivo*, treatment of healthy animals with fingolimod led to increased ERK1/2 phosphorylation and BDNF levels in cortical neurons. In the Rett disease model of MeCP2-deficient mice, a 4 week course of fingolimod treatment at a rather low dose (0.5 mg/kg every 4d) lead to increased levels of BDNF in affected brain structures and even to an amelioration of motor impairment and survival. This was the first description of a fingolimod-elicited production of the neurotrophin BDNF which could be attributed primarily to neurons by immunohistochemistry and cell culture experiments while a relevant contribution of astrocytes could be excluded (Deogracias et al., 2012).

## Epilepsy

Epilepsy is a chronic neurological disorder characterized by repetitive seizures, either of genetic (caused by mutations of genes encoding for excitatory or inhibitory signal tranducers in neurons or glial cells) or structural (caused by a brain lesion such as cerebral infarcts or brain tumors) origin. While epilepsies are well controllable by anticonvulsant medication in the majority of cases, series of recurrent seizures or non-terminating status epilepticus represent a serious threat for patients with epilepsy. The processes happening in the propagation from single seizures to prolonged epileptic activity have been characterized in experimental models and emcompass cell death, axonal and dendritic plasticity, neurogenesis and neuroinflammation (Lukasiuk et al., 2006). Especially neuroinflammatory processes such as glial cell activation and increases in inflammatory cytokines such as IL-1β and TNFα have recently gained interest (Ravizza et al., 2011; Vezzani and Friedman, 2011) and the blockade of IL-1β (Maroso et al., 2011) or TNFα (Rao et al., 2008) were shown to effectively decrease seizure activity. Gao et al. (2012) reported a significant decrease in the incidence and duration of spontaneous recurrent seizures by fingolimod treatment in rats that were subjected to an experimental model of lithium/pilocarpine-induced status epilepticus. Rats were treated with fingolimod 1 mg/kg daily starting 24 h after the induced status epilepticus and showed a reduced activation of microglia and astrocytes in the hippocampus in comparison to vehicle-treated mice as well as reduced hippocampal expression of the proinflammatory cytokines IL-1β and TNFα. Concerning a putative effect on neurons, there was abundant staining for the neuronal marker NeuN in immunohistochemical stainings of the hippocampus of healthy rats that was lost to a great degree 4 days after status epilepticus and partially rescued by FTY720 treatment. The loss of NeuN-positive Neurons in the hippocampus was accompanied by mossy fiber sprouting which was also reduced by treatment with FTY720.

Concerning putative non-immunomodulatory effects of FTY720 in epilepsy, it should be noted that mice with a genetic deletion of the S1P_2_ receptor develop spontaneous, sporadic, and occasionally lethal seizures between 3 and 7 weeks of age (MacLennan et al., 2001). At a cellular level, loss of the S1P_2_ receptor leads to a large increase in the excitability of neocortical pyramidal neurons, demonstrating that S1P_2_ plays an essential and functionally important role in the control of neuronal excitability.

## Brain tumors

Primary brain tumors most commonly originate from glial cells. Among them, high grade gliomas such as the glioblastoma multiforme (GBM) are the most malignant forms with aggressive growth and invasion of the surrounding brain tissue. Due to their infiltrating nature, they cannot be completely excised and the majority will recur locally (Giese et al., 2004). Life expectancy of patients diagnosed with GBM operated and treated with radiotherapy ranges around 12 months and can be extended by chemotherapy with the DNA alkylating chemotherapeutic temozolomid (TMZ), the current standard of care (Hart et al., 2013). There is increasing evidence that dysregulations of S1P metabolizing enzymes with increased levels of S1P are correlated with malignant properties of GBM. Van Brocklyn et al. (2005) showed that high expression levels of the SphK isoform SphK1 in human astrocytomas correlates with a 3fold shorter median survival of patients whereas in glioblastoma cell lines, RNA interference to knock down SphK2 even had a greater effect on tumor cell proliferation than knock down of SphK1 (Van Brocklyn et al., 2005). Exogenously added S1P has been found to stimulate motility and invasiveness of human GBM cells (Van Brocklyn et al., 2003). GBM cell lines express the S1P receptors S1P_1_, S1P_2_ and S1P_3_. From cellular the lower affinity S1P receptor ligands dihydro-sphingosine and sphingosylphosphorylcholine in the high nanomolar to low micromolar range, Van Brocklyn et al. (2002) concluded that the proproliferative effects of S1P on astrocytic tumor cell lines are mediated by the S1P1 receptor involving PI3K and ERK1/2 activation rather than by intracellular effects of S1P. This is supported by (partial) abolishments of S1P effects by the Gi protein inhibitor pertussis toxin or the phosphatidylinositol 3-kinase (PI3K) inhibitors wortmannin and LY294002 and the ERK1/2 kinase inhibiting MEK inhibitor U0126.

A thorough analysis of Young and Van Brocklyn (2009) on the contribution of the single S1P receptors to glioma cell proliferation, migration and invasiveness by means of differential overexpression or RNAi knockdown of the receptors on glioma cell lines established that S1P_1_, S1P_2_ and S1P_3_ all contribute positively to S1P-stimulated glioma cell proliferation, with S1P_1_ being the major contributor. Stimulation of glioma cell proliferation by these receptors correlated with activation of ERK1/2. Activation of S1P_5_ inhibited glioma cell proliferation and ERK1/2 activation. S1P_1_ and S1P_3_ enhance glioma cell migration and invasion. S1P_2_ inhibited migration through Rho activation, Rho kinase signaling and stress fiber formation, but enhanced invasiveness of glioma cells by stimulating cell adhesion. Thus, while S1P_2_ decreases glioma cell motility, it may enhance tumor cell invasion (Van Brocklyn et al., 2003; Lepley et al., 2005).

This may be in part mediated through induction of proteins that modulate glioma cell interaction with the extracellular matrix. S1P_2_ potently enhances expression of CCN1/Cyr61, a matricellular protein which stimulates cellular adhesion, migration, angiogenesis and invasion. A neutralizing antibody to CCN1 blocked S1P_2_-stimulated glioma invasion (Van Brocklyn et al., 2003). Expression of CCN1 has been shown to correlate with tumor progression and poor patient prognosis in glioma patients (Xie et al., 2004a) and to enhance tumorigenicity of glioma cells (Xie et al., 2004b). Besides that, uPA and its receptor uPAR, have been established as critical mediators of tumor cell invasiveness (Andreasen et al., 1997). In a subsequent study, Young et al. (2009) found that both CCN1 and uPA are upregulated in a GBM cell line upon treatment with S1P (100 nM). The expression of both proteins was induced through different individual S1P receptor subtypes that were overexpressed in a GBM cell line that normally expresses very low levels of S1P receptors. S1P_1_ and S1P_2_ receptors contribute to CCN1 induction while all three receptors, with S1P_1_ being the most potent, contribute to induce expression of members of the uPA system. Neutralizing antibodies directed against uPA or CCN1 significantly decreased both basal and S1P-stimulated GBM cell invasiveness. uPA activity and glioma invasion were potently blocked by SphK inhibition. Thus, the SphK/S1P/S1P receptor subtypes have a profound and coordinated effect on expression of several genes which are known to be involved in GBM invasiveness (Young et al., 2009).

Growth factors, including EGF, are known to stimulate SphK1 in several cell types and their overexpression in tumor cells is often associated with worse prognosis (Salomon et al., 1995). Especially the EGF receptor (EGFR) is often overexpressed and mutated in gliomas (Frederick et al., 2000). Estrada-Bernal et al. (2011) showed that treatment of glioma cell lines with EGF led to increases in SphK1 expression and activity. Expression of the constitutively active EGFRvIII mutant in glioma cells mimicked this effect. In addition, siRNA to SphK1 partially inhibited EGFRvIII-induced growth and survival of glioma cells as well as ERK1/2 activation. Interestingly, this effect could be overridden by treatment with high concentrations (10 μM) of S1P but not by lower concentrations. Since 10 μM S1P have been shown to activate the intracellular S1P target TRAF2, while lower concentrations of S1P (10 nM) are sufficient to activate the membrane-bound receptors, this hints to receptor-independent intracellular effects (Alvarez et al., 2010). Pharmacological blockade of the EGFR by gefetinib had a rather modest inhibitory effect on glioma cell proliferation whereas pharmacological inhibition of SphK1 strongly blocked proliferation and induced apoptosis of a GBM-derived neurosphere cell line (Estrada-Bernal et al., 2011). These data showed that EGF/EGFR and EGFRvIII signaling induces SphK1 in glioblastoma cells and that SphK1 is necessary to maintain survival of glioblastoma cells.

A putative therapeutic effect of fingolimod in the context of glioma research was evaluated as early as 2001 (Sonoda et al., 2001), spurred by the finding that higher concentrations of fingolimod in the micromolar range were shown to induce apoptosis in mature T lymphocytes (Enosawa et al., 1996). Sonoda et al. found that fingolimod at rather high concentrations (ED50 between 1–10 μg/ml) induced apoptosis in the human glioma cell line T89G. FTY720 led to tyrosine dephosphorylation of the focal adhesion kinase (FAK) and inhibited the protective FAK/PI3K pathway leading to an activation of caspase 6. Conversely, the inhibition of protein tyrosine phosphatases by orthovanadate prevented FAK dephosphorylation and inhibited fingolimod-induced cell death. An antitumor effect of fingolimod has been described in many tumor cell types (reviewed by Pitman et al., 2012; Zhang et al., 2013). In most cases, these antitumor effects are caused by unphosphorylated fingolimod, not fingolimod phosphate in cell culture systems and seem to involve direct intracellular effects of fingolimod such as an activation of phosphatase 2A (PP2A), the tumor suppressor PTEN, an inhibition of the PI3K/AKT/mTOR pathway or even an inhibition of SphKs. Estrada-Bernal et al. (2012) reproduced the propapoptotic effect of fingolimod on glioma cells in the pathophysiologically more relevant model of brain tumor stem cells (BTSCs) derived from human GBM tissue. These cells develop neurosphere-like aggregates in cell culture and develop into faithful histological models of human gliomas when injected into the brains of mice. Four different BTSC lines underwent apoptosis at FTY720 concentrations of 1 μg/ml and higher whereas toxicity to non-malignant primary astrocytes began at 10 μg/ml and was thus one order of magnitude higher. Fingolimod led to a rapid dephosphorylation of ERK1/2, upregulation of the BH3-only protein Bim, and cleavage of caspases 9 and 7 or caspase 3. Fingolimod also reduced BTSC invasiveness and, most importantly, had a synergistic effect in addition to TMZ, the current standard chemotherapeutic agent to treat malignant gliomas, allowing a reduction of the fingolimod dose required for BTSC toxicity. By contrast, fingolimod phosphate was much less effective at inducing apoptosis of BTSC. Generally, in animal models of many cancers, exceedingly high FTY720 doses of 5–10 mg/kg are required for satisfying effectiveness, as compared to 0.5–1 mg/kg that were found to be effective in models of cerebral ischemia (Czech et al., 2009; Hasegawa et al., 2010; Wei et al., 2011; Kraft et al., 2013) or neurodegeneration (Deogracias et al., 2012; Takasugi et al., 2013) and even lower doses required in EAE (Brinkmann et al., 2002). This was also described in the experimental glioma model of Estrada-Bernal et al. (2012), who injected glioma BTSCs into nude mice that were treated with either fingolimod (10 mg/kg), TMZ (5 mg/kg) or a combination of both drugs. This experiment confirmed the efficacy of fingolimod as an experimental glioma therapy that significantly prolonged survival and reduced tumor growth even though it was somewhat less effective than TMZ. The combination of both drugs showed the greatest efficacy. Even if due to yet undefined reasons, the *in vivo* effect of fingolimod was somewhat weaker than the stunning *in vitro* effects, fingolimod seems to be a promising (adjuvant) drug to treat gliomas, whose progression is yet barely controlled by the current standard therapies.

## Summary and future perspectives

According to experimental data, the sphingolipid signaling pathway seems to be of central relevance in the pathophysiology of many diverse neurological diseases. The versatile sphingosine analog fingolimod, prodrug of the S1P-modulating fingolimod phosphate, provides scientifically proven reduction of relapse rate and amelioration of disease progression in MS. But data from experimental animal models suggest that fingolimod has additional therapeutic applications in store. Many of the diseases reviewed here follow a fatal course and treatment options yet are scarce and their efficacy often short-lived. Given the growing body of experience with fingolimod accumulated by clinical neurologists and the favorable safety profile in the indication of MS, there is hope for a translation of some of these experimental findings into the clinics.

## Conflict of interest statement

The authors declare that the research was conducted in the absence of any commercial or financial relationships that could be construed as a potential conflict of interest.
